# Supplementary material for: Patterns of Intron Gain and Loss in Fungi
Source: PLoS Biol. 2004 Nov 30;2(12):e422. doi: 10.1371/journal.pbio.0020422 (PMC532390; doi:10.1371/journal.pbio.0020422)
Supplement: Table S1 — Also available at http://genes.mit.edu/NielsenEtAl/. (4.3 MB ZIP). [file pbio.0020422.st001.zip › NielsenEtAl/html/49.html]

AN6338.1.NCU09116.1.MG09919.1.FG01285.1


```
 CLUSTAL W (1.82) Multiple Sequence Alignments - Introns Inserted


Sequence 1: NCU09116.1	560 aa
Sequence 2: MG09919.1	587 aa
Sequence 3: FG01285.1	559 aa
Sequence 4: AN6338.1	529 aa
Alignment Length: 590 aa
Number Identitical Residues: 198 aa
Alignment Score (without introns) 11532


MG09919.1 	MSTSTQVRTSSAIKIMSPTLLAIDESAAAAETVGPIGLPAKQVPEHPNSVAARAPKLERP
NCU09116.1	-MGEALINGSGAVSNVS-----------ALETLG----PAAKLTDADPSEIPPASGPGRE
FG01285.1 	--MLSAVRTARLRSLIK-----------HSRTIHS---SSKHLSSTEAAPAARPAHYDTS
AN6338.1  	-------------------------------------------MSPPAALDMDTNGIGAP
          	                                            .   :    .      

MG09919.1 	RRIT-FKDIADKRTRAGKLVALTASASDSDMFKVA~AAGQKPKARRWDH~LLSQESASRK
NCU09116.1	NAVT-ISDILERRAKAGRLIAPTASYADSDMFKGP0QVG-KPKSKSMNH1HLSAESLSRH
FG01285.1 	QKLSAIDAVKERRLKAGKLVAGVAAASDSDMFKGP0TTG-LPKSKRWDN1HLSHESKVRE
AN6338.1  	KPLT-VDGIAAVRAKSAPMPAGVAPATSSDMFKSP0ACYTKPKAKRWDN1FFTLEAKSRK
          	. :: .. :   * ::. : * .*. :.***** .      **::  ::  :: *:  *.

MG09919.1 	PCRLKQAALNLRKPG~IISLGGGLPCPDNFPIESISMRVPSVSGGFSEADTRAHGLDVTV
NCU09116.1	PCALKQAARHLKNPN~IISLGGGLPCAEYFPIESITLKVPTPP-DFSEAATSQSGAEVRI
FG01285.1 	PCTLKQAARHMKKPG~LISLGGGLPSSEVFPFAELGFKVPVAP-KFSEKDTEESGQTVTI
AN6338.1  	TSTLKGAAKFLKNPG1LISLGGGLPSPEYFPFEELSFKVPTPP-GFSPEATRQSGTTLVA
          	.. ** **  :::*. :********..: **: .: ::**  .  **   *   *  :  

MG09919.1 	GKYDVQEQAEDPNGAVYDLSIALNYSQATGSAQMMRFVTEHTE~LVNNPPYADWRCSMSI
NCU09116.1	GKYDATDPSIQG---TYDLAVALNYAQASGSAQLLRFVTEHTE~LVHNPPYSDWRSCLTI
FG01285.1 	GKYDVRDR-----GGTYDLSIACNYGQATGSPQMMRYLTEHTE~IVYKPPYADWKVCQTI
AN6338.1  	GKHDIREGK-----SLYDLEVSLNYGQSTGSPQLLRFVTEHTE0LIHNPPYSDWQCALTA
          	**:*  :       . *** :: **.*::**.*::*::***** :: :***:**: . : 

MG09919.1 	GSTGALEMALRIFCDR-ARNDSMLTEEFSFSTAVETAMPLGIKVFGVPVDGEGLIPEKMD
NCU09116.1	GSTGALEQTLRMLCDGPGRRDSLLTEEYSFSTALETAGPLDLKVVGVKMDGQGLLPEAMD
FG01285.1 	GSTGALEEALRMFCDK-DRGDSVLTEDFSFSTALETVGPLGIKAFGVSIDEQGLIPEAMD
AN6338.1  	GSTYAWDTALRLFCER---GDYLLMEEYTFSSAQETALPQGVNVAPVKMDEQGLLPGDLD
          	*** * : :**::*:     * :* *:::**:* **. * .::.  * :* :**:*  :*

MG09919.1 	EILRTWKPEERKGAAKPHVLYTVPSGQNPTGSTQSEERRRALYQVCREHDVYIIEDEPYY
NCU09116.1	ELLSNWNPEEREGRRKPHVLYTVPSGQNPTGTTQGLERRKQVYEVARKHDLFVIEDEPYY
FG01285.1 	ELLSNWDAKER-GSRKPHLLYTVPSGQNPTGATQGTERRKAIYAVAQKHDIYIIEDEPYY
AN6338.1  	DVLSNWDEKVR-GARKPFVLYTIPTGQNPTGATQSAERRKEIYKVAQKHNIYIIEDEPYY
          	::* .*. : * *  **.:***:*:******:**. ***: :* *.::*::::*******

MG09919.1 	YIQMPPYRGRDAKKEEEQSNDAEETPEQFLESLVPSILSMDVDGRVLRMDSFSKVVVPGS
NCU09116.1	YLQMP-------LEESKETESEEETVESFMSSLIPSLLSLDVDGRVLRMDSFSKVVVPGS
FG01285.1 	FLQMQPY-----TGRNQPDVPPPKTVDEFVSSLIPSLLSIDVDGRVMRMDSFSKVLVPGS
AN6338.1  	FLQMQPY-----TGADSAPVPPPANHEEFVKSLIPSYLSIDVDGRVLRVESFSKVLAPGS
          	::** .         ..       . :.*:.**:** **:******:*::*****:.***

MG09919.1 	RMGWVTASEQIIDRFIRHGESCNQGPSGIAQVLLHKLLDETWGHEGYIRWLMSVRGEYTR
NCU09116.1	RVGWVTASENIIEQYLRHAEVCNQGANGISQVILYKLLDEQWGHEGYFKWLMNLRQEYTK
FG01285.1 	RLGWITASEQIVERYIRHAEVASQGPSGFSQVILYKLLDETWGHEGYLRWLMNLRLEYTK
AN6338.1  	RTGWVVGSEQVIERFLRNTEVSAQHPSGISQLMLFKLLDEHWGHLGYFDWLINLRMQYTN
          	* **:..**:::::::*: * . * ..*::*::*.***** *** **: **:.:* :**.

MG09919.1 	RRDVLLAACEDHLPREVVNWEVPKTGMF0HWLKVDHTKHP-CASTMSISEMEAEIFKDCI
NCU09116.1	RRDTMVTAAEKFLPKEVVSWTVPVAGMF0YWLKIDHSQHPGIQAGKGILEVEEEIFNSCI
FG01285.1 	KRDALLAACEDHLPSDLASWTPPVAGMF0MWINIDYTKHP-DAGKRSIVDIEEEIFNLCI
AN6338.1  	RRNIMVQACEKHLPQEIASWIAPAAGMF0HWIQVDWKKHPELTSGKTRDSIEEALFHAAI
          	:*: :: *.*..** ::..*  * :***  *:::* .:**   .     .:*  :*: .*

MG09919.1 	DKGVLVAAGSWFMADHDAEPSGLFFRATFAAATPDNMIEATRRLGDAIRSSFQLN
NCU09116.1	ARGVLVARGSWFRAEQDTEPNELFFRATFAAATAENMTEAIRRFGEAIRESFKL-
FG01285.1 	ENGVLIARGSWFLTEKDKAPPGLFFRATYASATPENMNKAIERFGKAVRDSFGRK
AN6338.1  	NNGVLVSRGSWFKAAGASE-DKLFFRATFAAASEENITEAIRRFGDALRVEFKL-
          	 .***:: **** :        ******:*:*: :*: :* .*:*.*:* .*
```
